# The C: N: P stoichiometry in bryophytes: relationships with habitat, climate and growth form

**DOI:** 10.1093/nsr/nwad060

**Published:** 2023-03-06

**Authors:** Xin Liu, Zhe Wang, Xiaoming Li, Weikai Bao, Kathrin Rousk

**Affiliations:** China-Croatia ‘Belt and Road’ Joint Laboratory on Biodiversity and Ecosystem Services, CAS Key Laboratory of Mountain Ecological Restoration and Bioresource Utilization, and Ecological Restoration and Biodiversity Conservation Key Laboratory of Sichuan Province, Chengdu Institute of Biology, Chinese Academy of Sciences, China; Shanghai Normal University, China; China-Croatia ‘Belt and Road’ Joint Laboratory on Biodiversity and Ecosystem Services, CAS Key Laboratory of Mountain Ecological Restoration and Bioresource Utilization, and Ecological Restoration and Biodiversity Conservation Key Laboratory of Sichuan Province, Chengdu Institute of Biology, Chinese Academy of Sciences, China; China-Croatia ‘Belt and Road’ Joint Laboratory on Biodiversity and Ecosystem Services, CAS Key Laboratory of Mountain Ecological Restoration and Bioresource Utilization, and Ecological Restoration and Biodiversity Conservation Key Laboratory of Sichuan Province, Chengdu Institute of Biology, Chinese Academy of Sciences, China; Department of Biology, Terrestrial Ecology Section, University of Copenhagen, Denmark

The elemental composition and stoichiometry of organisms provide a useful lens for explaining and predicting ecological patterns and processes. However, few studies have focused on stoichiometry in bryophytes, even though they are abundant in many pristine ecosystems. Several case studies found variable patterns in bryophyte stoichiometry (e.g. [1,2]). Yet, the large-scale stoichiometric pattern for bryophytes and whether the pattern coincides with the predictions from general stoichiometric hypotheses is largely unknown.

One contributor to stoichiometric variations is environmental nutrient availability (Fig. 1A). The temperature-biogeochemistry hypothesis predicts plant N and P would increase with temperature [3], as cold temperatures limit metabolic activities of microbes that decompose organic matter, and thereby reduce the availability of N and P. The hypothesis assumes that plants mainly rely on one nutrient source, e.g. soil nutrient pools. Yet, bryophytes can take up nutrients from multiple sources [1], and soil N only accounts for ∼37% of bryophyte N. Thus, the temperature-biogeochemistry hypothesis may not apply to bryophytes. On the other hand, having thin leaves and no cuticular barrier in most taxa, bryophytes can have various environmental nutrient sources. Differences in the physical and biological characteristics of terrestrial and aquatic habitats result in differences in nutrient availabilities, leading to different stoichiometry.

**Figure 1.**
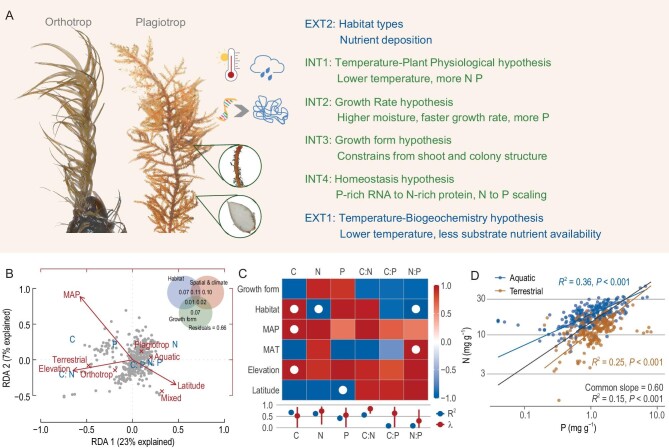
Bryophyte C: N: P stoichiometry and affecting factors. (A) Hypotheses and theories relate to elemental concentration and stoichiometry in bryophytes. Pictures of orthotropic moss, *Bartramia halleriana*, and plagiotropic moss, *Thuidium cymbifolium*, are on the left side. Hypotheses related to external nutrient availability are labeled with ‘EXT’, while theories related to physiological and structural constraints are labeled with ‘INT’. (B) Redundancy analysis (RDA) of C, N and P concentrations and their ratios of bryophytes. The RDA model was built by stepwise model selection using permutation tests. Explanatory variables are habitat, growth form, MAP, latitude and elevation. Variation partitioning of bryophyte stoichiometry explained by three groups of factors is in the right corner. The value in each fraction indicates the explained variation (*P* < 0.001). Residuals indicate unexplained variation. See Table S2 for further details. (C) Relationships of bryophyte elemental composition (columns) with growth form, habitat types, and spatial and climatic factors (rows). Colors indicate the probability of direction (PD) of the posterior distributions of the relationship between pairs of variables according to the scale bar reported at the right. Dots indicate Bayes factors > 1. The amount of variance explained (R^2^) and the phylogenetic signal (Pagel's λ) and 95% credible intervals are in the bottom panel. See supplementary Appendix 2 for model summaries. (D) Scaling relationship between N and P concentrations according to standardized major axes (SMA) regression. See Table S3 for further details.

The stoichiometric variation is also likely controlled by physiological, structural and phylogenetic constraints. Plants adapted to cold habitats, as predicted by the temperature-plant physiology hypothesis, may contain greater N and P than those grown in warm environments [3], and thus compensate for the influence of low temperature on N and P regulated metabolic processes. Bryophytes, despite limited empirical evidence, should follow the temperature-plant physiology hypothesis, as they share mostly the same physiological processes as vascular plants. The growth-rate hypothesis predicts that plants with faster growth rates require higher amounts of P-rich RNA [4]. Moisture conditions, a main regulator of plant growth, strongly influence the growth rate of bryophytes, as they generally lack highly developed vascular systems. Thus, the stoichiometry for bryophytes should relate to moisture.

Evolutionary history imposes taxon-specific constraints on stoichiometric variation, resulting from phylogenetically conserved differences among shoot or colony structure, innate growth rate and ecological strategy. Bryophyte growth forms, on the other hand, are largely genetically controlled, and represent not only adaptation but also resource allocation strategies. Bryophytes with different growth forms (Fig. 1A) have erect or prostrate shoots comprised of different proportions of leaves and stems. Leaf nutrient concentrations are in contrast to those of a stem because of their different structure and function. Thus, growth form should affect bryophyte stoichiometry.

Bryophytes are assumed to have weak homeostatic regulations as they are sensitive to environmental nutrient conditions. Yet, studies found evidence for stoichiometric homeostasis [1,2]. Furthermore, whether bryophytes have homeostatic regulation, and whether they follow the general 2/3-power law of N to P scaling found in vascular plant leaves, is an open question considering their differences in nutrient uptake and use.

Herein, we examine the stoichiometric pattern and its potential drivers for bryophytes. To that end, we searched Web of Science and China National Knowledge Infrastructure for relevant articles and extracted C, N and P concentrations and their ratios, as well as latitude, longitude, mean annual temperature (MAT), mean annual precipitation (MAP), habitat type, species identity, growth form and life form if available. We built a phylogeny covering 139 bryophytes based on the trnL gene and performed a mixed multivariate and phylogenetically informed model using a Bayesian framework. In addition, we performed regressions, redundancy analysis (RDA) and variance partitioning to examine the effects of habitat types, climate and growth forms, since species identities were not available for some of the data and could not be incorporated in the phylogenetic analysis (see supplementary Appendix 1 for details).

Our estimate of C: N: P stoichiometry for bryophytes was 923:31:1 on a molar basis. The estimation diverged from the Redfield's ratio of 106:16:1 for marine plankton and the ratios of 550:30:1 for benthic marine plants. But the C: N: P ratio for bryophytes was similar to the estimation for terrestrial plant leaves of 799:27:1. The data revealed large variations in the stoichiometry of bryophytes (Table S1). The RDA and variance partitioning showed that ∼30% of the variations were explained by environmental factors, innate characteristics of bryophytes, such as growth form, and their interactions (Fig. 1B). The variance explained by phylogenetically informed models was higher for C (0.67), N (0.62), P (0.42) and C: N (0.56) than for C: P (0.08) and N: P (0.09). The median phylogenetic signal ranged from 0.30 to 0.83 (Fig. 1C).

We found contrasting results between the relationships of N and P versus MAT. For MAT < 16°C, the generally increasing N content of bryophytes with increasing MAT (Fig. S1) appeared to contradict the temperature-plant physiology hypothesis that nutrient concentration should decrease with increasing temperature. The increasing N with MAT is probably due to bryophyte-associated N_2_ fixation, as N_2_-fixation activity is positively related to temperature and the optimum temperature is 16–27°C [5]. For temperatures < 12°C, the increasing P and decreasing C: P with decreasing MAT is consistent with the temperature-plant physiology hypothesis implying a cold tolerance mechanism. Both phylogenetic and quadratic models showed positively related N: P and MAT (Fig. 1C and Fig. S1). The relation, however, cannot be ascribed to soil nutrient availability as in vascular plants [3], because most bryophytes are less dependent on soil nutrient pools [1] and analysis of aquatic bryophytes shows similar results. Besides, P deposition has no clear spatial pattern. Thus, bryophytes appear to incorporate more P at low temperatures and fix more N at intermediate temperatures.

The increasing P and decreasing N: P with MAP for sites with precipitation < 1900 mm supported the theory that P should increase with MAP for faster growth rates [4]. However, the phylogeny-informed models found no significant relationship between N, P and N: P versus MAP, which suggests species turnover along the precipitation gradient (Fig. 1C and Fig. S2).

Both phylogenetic and non-phylogenetic models found significant effects of habitat on stoichiometric ratios (Fig. 1C and Fig. S3). Terrestrial bryophytes had higher C concentration and C: N likely due to higher demand for more C-rich supportive structures and the requirement of dense colonies to keep moist [2]. On the other hand, aquatic bryophytes, with lower C: N than terrestrial bryophytes, are less limited by hydraulic and mechanical constraints, can optimize area-based functions, such as light-harvesting [6], and result in higher N than terrestrial species.

Regressions and RDA showed significant effects of growth form on bryophyte stoichiometry (Fig. 1B, Figs S4 and S5). Orthotrops allocate more resources to C-rich structural components. The higher N and P in plagiotrops than in orthotrops confirmed that morphological plasticity is a resource acquisition mechanism, as plagiotrops can alter colony structure and surface area by changing branching patterns. However, phylogeny-informed models failed to find an effect of growth form (Fig. 1C, Fig. S5 and supplementary Appendix 2). The results suggest that stoichiometry is, to a certain extent, genetically determined, as growth form is associated with phylogeny. Furthermore, variance partitioning found that the effect of growth form was largely independent of habitat and climate (Fig. 1B) and confirmed the genetically determined stoichiometry leading to stoichiometric homeostasis.

Bryophyte N was positively correlated to P (Fig. 1D), indicating stoichiometric homeostasis [3,4]. Considering that bryophytes are largely dependent on nutrients from highly variable atmospheric deposition, our result suggests that bryophytes actively control their nutritional balance and their ability to translocate or resorb nutrients might be a universal feature [7] underpinning the N to P scaling. The scaling exponent for terrestrial bryophytes, 0.68, was remarkably similar to the value of 0.676 reported as general for vascular plant leaves [8] and significantly higher than that for aquatic bryophytes (0.40, *P* < 0.001, Table S3). The N to P scaling for vascular plant leaves is closely associated with relations of mass-based nutrient concentration to specific leaf area [8], which involve area–mass relations depending on the quantity of hydraulic or mechanical tissues in the lamina. In most bryophytes, however, the structure of colony determines the area–mass relation, as it is colony structure that controls water retention in capillary spaces [9] and light interception [6]. Canopy mass per area, a parameter depicting bryophyte colony structure, correlated to photosynthetic rate is analogous to the relations found for the leaf economic spectrum [10], while no relationship was found at the shoot level. The requirements for water retention and structural support for an aquatic bryophyte differed from those for a terrestrial bryophyte, leading to contrasting colony structures and area–mass relations, and thus distinct N to P scaling exponents.

The bryophyte stoichiometric variation results from several drivers including nutrient availability, adaptation and acclimation of physiological processes to thermal and moist environments, and phylogenetic and structural constraints. The results could not confirm the temperature-biogeochemistry hypothesis that predicts increasing N and P contents with increasing temperature. Our current study provides novel insights into bryophytes’ nutrient use strategy and a basis for further research on biogeochemistry and ecosystem functioning.

## Supplementary Material

nwad060_Supplemental_File
