# Supplementary material for: The C: N: P stoichiometry in bryophytes: relationships with habitat, climate and growth form
Source: Natl Sci Rev. 2023 Mar 6;10(4):nwad060. doi: 10.1093/nsr/nwad060 (PMC10042222; doi:10.1093/nsr/nwad060)
Supplement: nwad060_Supplemental_File [file nwad060_supplemental_file.docx]

**SUPPORTING INFORMATION FOR**

**The C:N:P stoichiometry in bryophytes:
relationships with habitat, climate and growth form**

Xin Liu^1,#^, Zhe Wang^2,#^, Xiaoming Li^1^, Weikai Bao^1,*^, Kathrin Rousk^3^

Affiliations:

^1^ China-Croatia "Belt and Road" Joint Laboratory on Biodiversity and Ecosystem Services, CAS Key Laboratory of Mountain Ecological Restoration and Bioresource Utilization & Ecological Restoration and Biodiversity Conservation Key Laboratory of Sichuan Province, Chengdu Institute of Biology, Chinese Academy of Sciences, Chengdu 610041, China

^2^ Shanghai Normal University, Shanghai 200234, China

^3^ Department of Biology, Terrestrial Ecology Section, University of Copenhagen, Universitetsparken 15, 2100 Copenhagen, Denmark

Figure S1


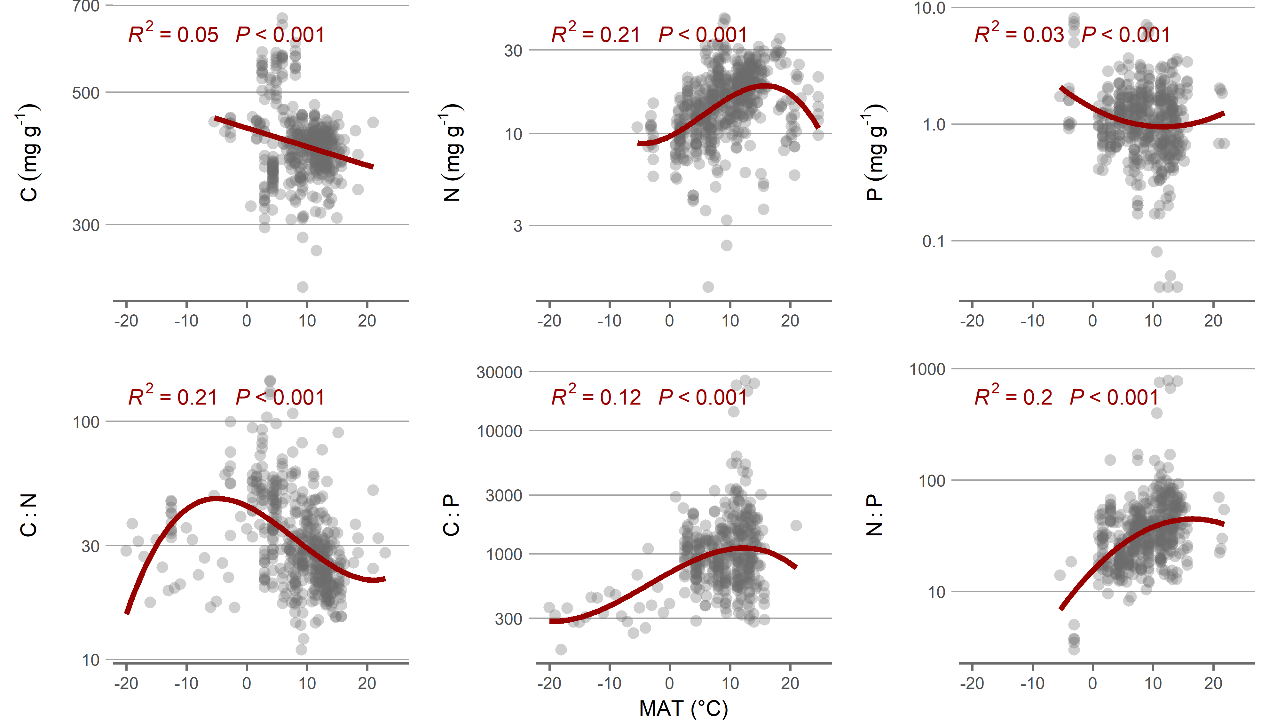


Figure S1 Carbon (C), nitrogen (N), phosphorus (P) concentrations and their stoichiometric ratios (C:N, C:P and N:P) of bryophytes in relation to mean annual temperature (MAT).

Figure S2


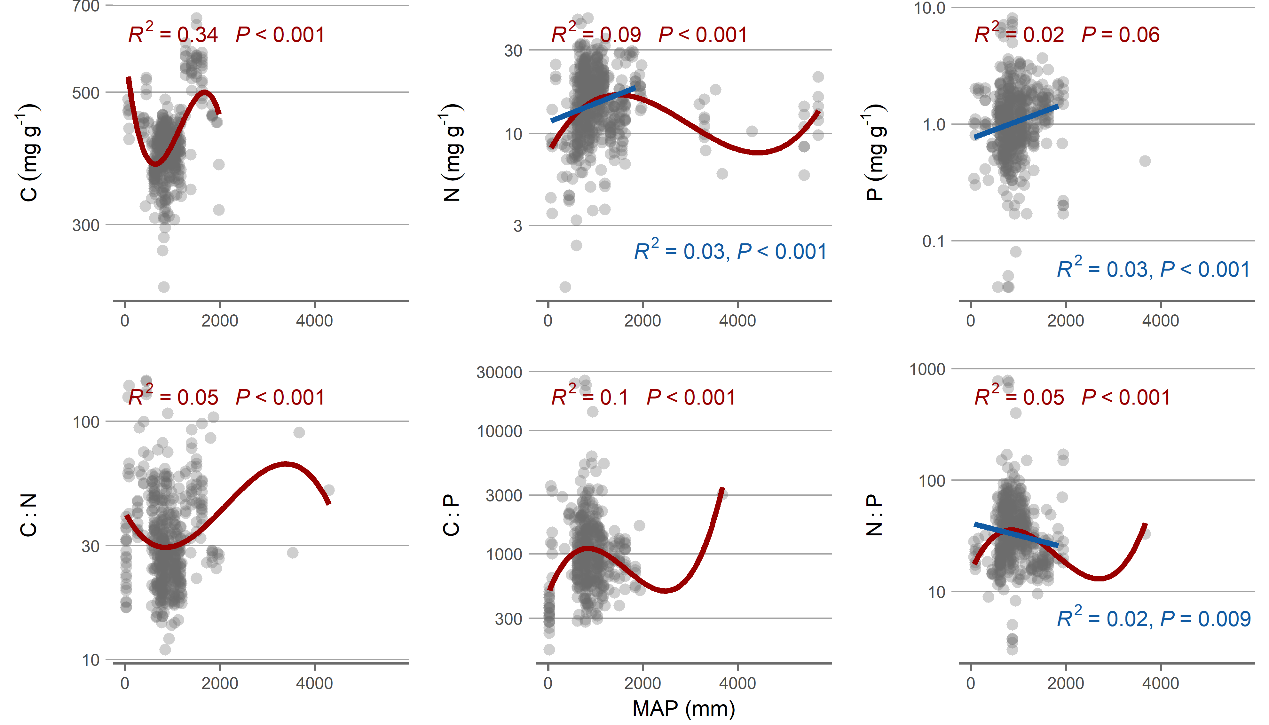


Figure S2 Carbon (C), nitrogen (N), phosphorus (P) concentrations and stoichiometric ratios (C:N, C:P and N:P) of bryophytes in relation to mean annual precipitation (MAP). The red line and its statistics (R^2^ and P value) presents the relationship between elemental concentrations or ratios and MAP for all observations, while the blue line presents the relationship between nutrient concentrations or ratios and MAP for those sites with precipitation less than 1900 mm.

Figure S3


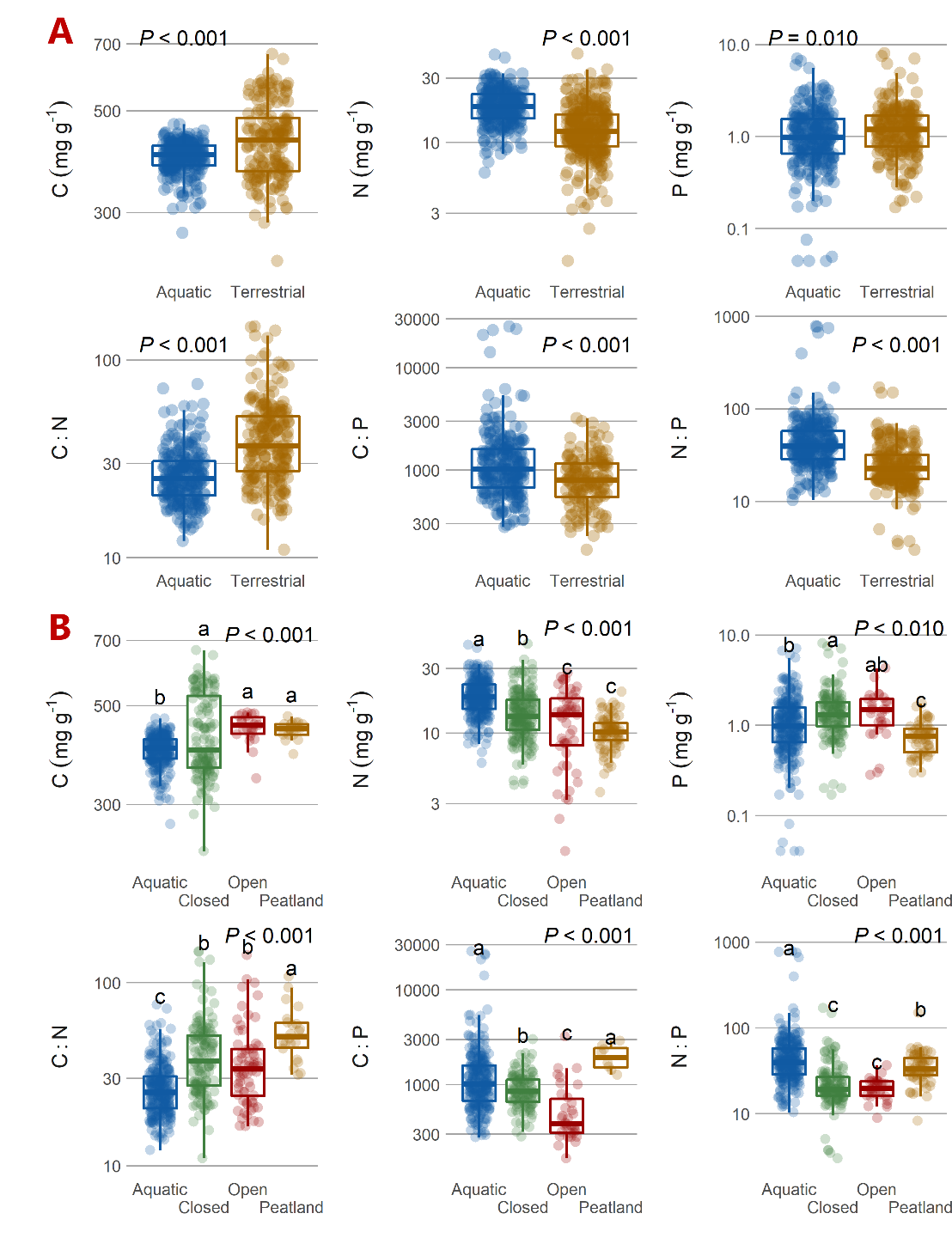


Figure S3 Carbon, nitrogen and phosphorus concentrations and their ratios for bryophytes between aquatic vs terrestrial habitats (A), and among aquatic and 3 terrestrial habitats (closed habitats such as forests, open habitats such as deserts and tundra and peatland, B). P values represent the significances of bootstrap t test (A) and one way ANOVAs (B). Letters show significance according to Tukey’s HSD test (P <0.05).

Figure S4


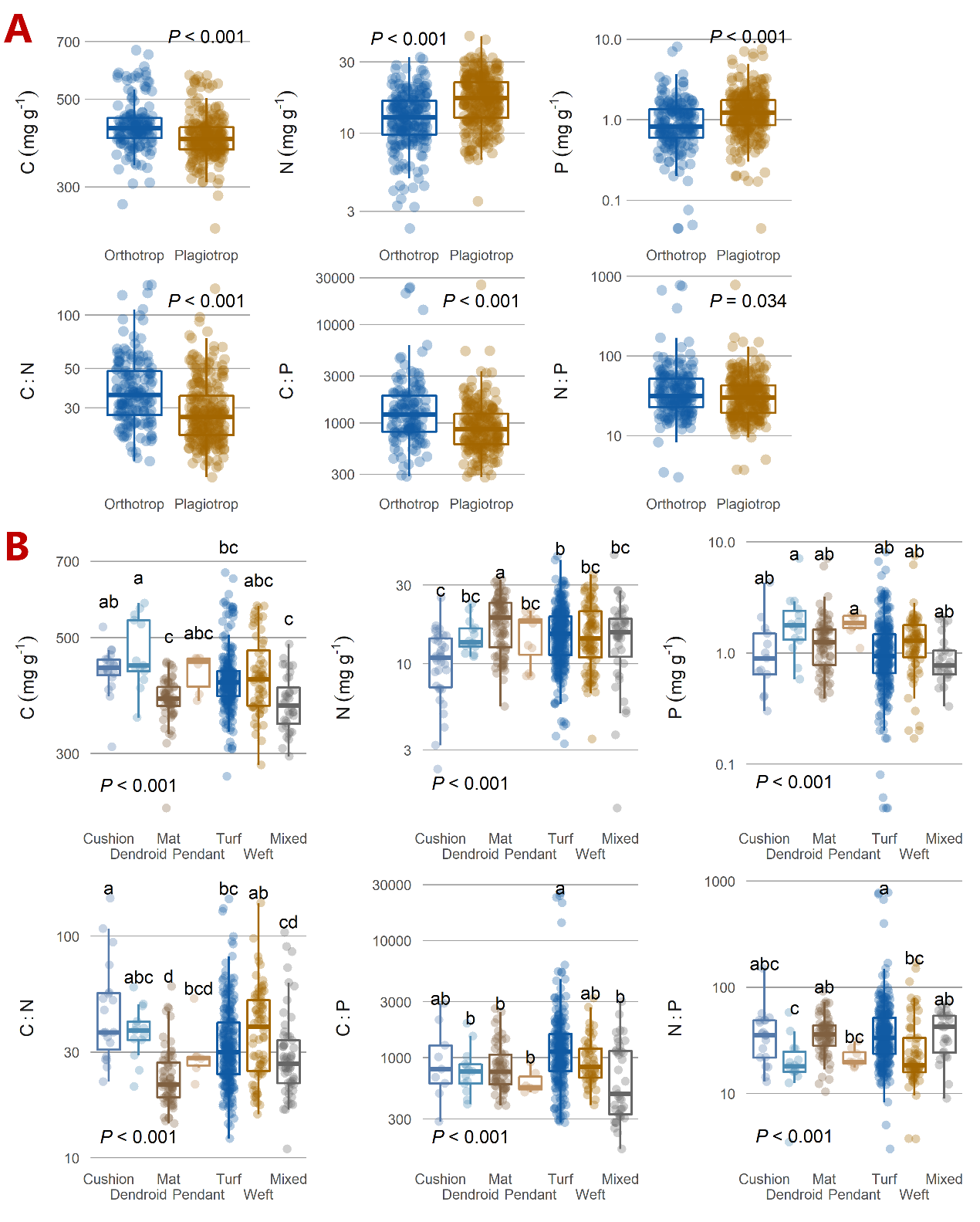


Figure S4 Carbon, nitrogen and phosphorus concentrations and their ratios between bryophytes with different growth froms (A) and life forms (B). P values represent the significances of bootstrap t test (A) and one way ANOVAs (B). Letters show significance according to Tukey’s HSD test (P <0.05).

Figure S5


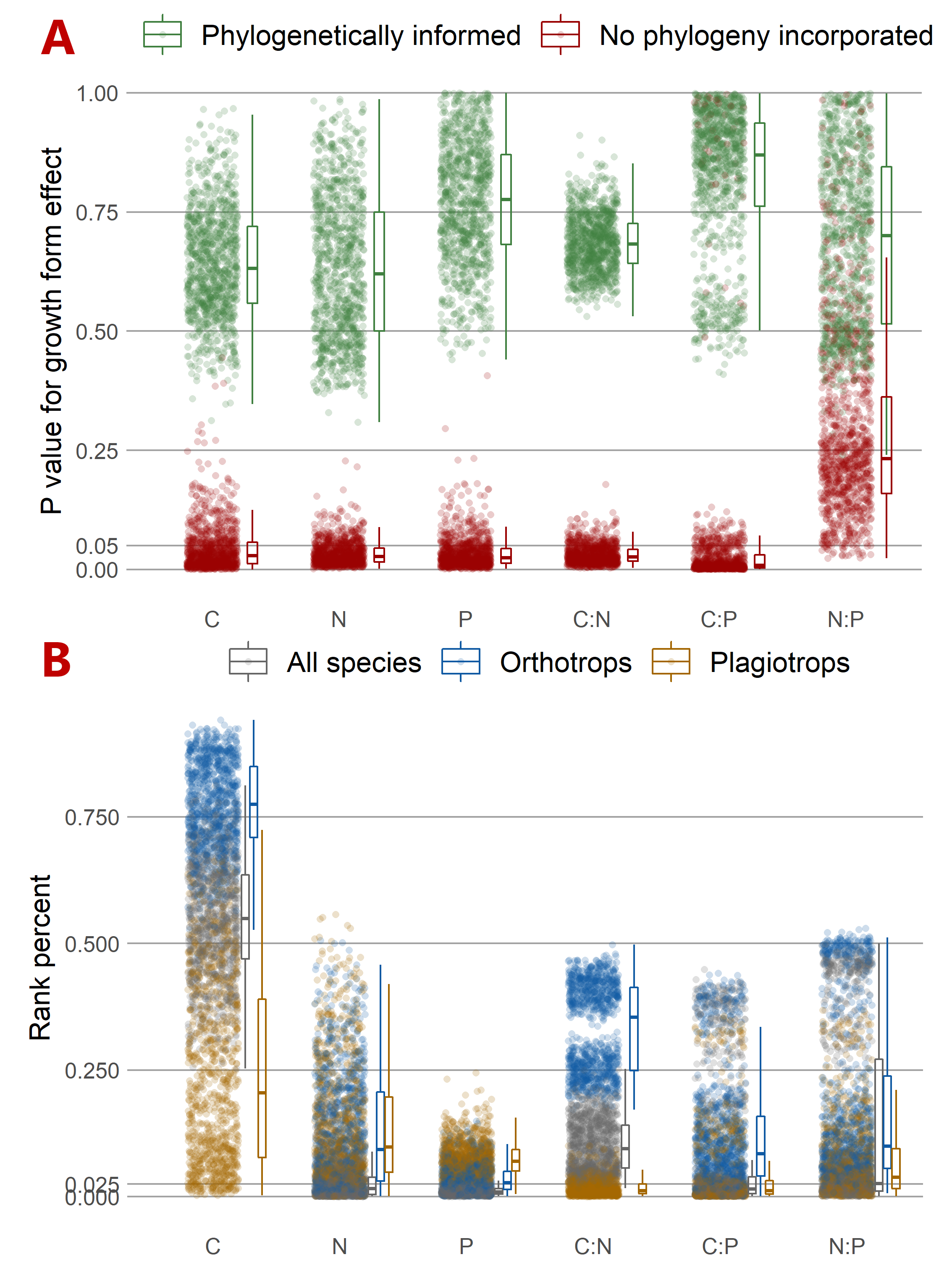


Figure S5 The effects of growth form (A) according to phylogenetically informed and no phylogeny incorporated model; and the rank values (B) of mean phylogenetic independent contrasts (PICs) among random null distributions for orthotrops, plagiotrops and all species. The results indicated significant effects of growth form on bryophyte stoichiometry, while the effects can also be explained by phylogeny as no significant effect of growth form was found in the phylogenetically informed model, suggesting growth form was associated with phylogeny. However, growth form cannot explain all the phylogenetic effects because of significant phylogenetic signals in C:N and C:P for orthotrops.

Table S1

Summary of bryophyte carbon (C, mg g^-1^), nitrogen (N, mg g^-1^), phosphorus (P, mg g^-1^) concentrations and their molar ratios. Number of observations (No.), minimum (Min.), maximum (Max), median, coefficient of variation (CV, %), mean and standard error (SE) were shown. P values indicate significance of bootstrap t-test between habitats (Aquatic vs Terrestrial) and between growth forms (Orthotrop vs Plagiotrop).

|  | Group | No. | Min. | Max. | Median | CV(%) | Mean | SE | P |
| --- | --- | --- | --- | --- | --- | --- | --- | --- | --- |
| C | All | 425 | 235.50 | 665.53 | 409.25 | 15 | 414.03 | 2.96 | - |
|  | Aquatic | 247 | 271.15 | 467.58 | 401.73 | 8 | 397.44 | 2.01 | <0.001 |
|  | Terrestrial | 178 | 235.50 | 665.53 | 436.2 | 19 | 437.04 | 6.10 |  |
|  | Orthotrop | 156 | 271.15 | 665.53 | 423.58 | 15 | 435.06 | 5.28 | <0.001 |
|  | Plagiotrop | 233 | 235.50 | 575.4 | 397.77 | 13 | 405.77 | 3.58 |  |
| N | All | 574 | 1.33 | 45.60 | 15.16 | 41 | 15.90 | 0.27 | - |
|  | Aquatic | 257 | 5.99 | 44.81 | 18.51 | 30 | 19.11 | 0.36 | <0.001 |
|  | Terrestrial | 317 | 1.33 | 45.60 | 12.12 | 45 | 13.31 | 0.33 |  |
|  | Orthotrop | 221 | 2.30 | 32.30 | 12.78 | 40 | 13.37 | 0.36 | <0.001 |
|  | Plagiotrop | 310 | 3.50 | 44.81 | 17.26 | 37 | 17.74 | 0.37 |  |
| P | All | 480 | 0.04 | 8.07 | 1.08 | 78 | 1.30 | 0.05 | - |
|  | Aquatic | 255 | 0.04 | 7.06 | 0.98 | 78 | 1.23 | 0.06 | 0.010 |
|  | Terrestrial | 225 | 0.17 | 8.07 | 1.19 | 77 | 1.37 | 0.07 |  |
|  | Orthotrop | 182 | 0.04 | 8.07 | 0.82 | 85 | 1.10 | 0.07 | <0.001 |
|  | Plagiotrop | 273 | 0.04 | 7.48 | 1.23 | 73 | 1.46 | 0.06 |  |
| C:N | All | 473 | 10.97 | 148.17 | 28.84 | 56 | 35.04 | 0.91 | - |
|  | Aquatic | 247 | 12.19 | 75.76 | 25.23 | 36 | 27.24 | 0.62 | <0.001 |
|  | Terrestrial | 226 | 10.97 | 148.17 | 37.19 | 55 | 43.57 | 1.59 |  |
|  | Orthotrop | 164 | 15.03 | 148.17 | 35.59 | 55 | 41.55 | 1.77 | <0.001 |
|  | Plagiotrop | 252 | 12.19 | 141.17 | 26.79 | 53 | 31.46 | 1.05 |  |
| C:P | All | 401 | 166.63 | 25405.33 | 928.64 | 174 | 1406.95 | 122.51 | - |
|  | Aquatic | 247 | 278.97 | 25405.33 | 1022.18 | 181 | 1693.62 | 194.63 | <0.001 |
|  | Terrestrial | 154 | 166.63 | 3533.09 | 807.18 | 62 | 947.16 | 47.43 |  |
|  | Orthotrop | 138 | 284.08 | 24032.21 | 1214.42 | 172 | 1998.22 | 292.13 | <0.001 |
|  | Plagiotrop | 224 | 278.97 | 25405.33 | 866.92 | 155 | 1143.86 | 118.48 |  |
| N:P | All | 479 | 3.00 | 779.65 | 30.98 | 161 | 43.72 | 3.22 | - |
|  | Aquatic | 255 | 10.36 | 779.65 | 39.73 | 161 | 57.53 | 5.80 | <0.001 |
|  | Terrestrial | 224 | 3.00 | 170.1 | 22.73 | 71 | 28.01 | 1.33 |  |
|  | Orthotrop | 186 | 3.00 | 771.18 | 31.62 | 182 | 51.95 | 6.92 | 0.034 |
|  | Plagiotrop | 267 | 3.72 | 779.65 | 30.08 | 133 | 38.40 | 3.13 |  |

Table S2

Permutation test of explanatory variables for the RDA model.

|  | df | Variance | F | P |
| --- | --- | --- | --- | --- |
| Habitat | 1 | 0.6225 | 49.8604 | < 0.001 |
| MAP | 1 | 0.3349 | 26.7490 | < 0.001 |
| Latitude | 1 | 0.2915 | 23.2783 | < 0.001 |
| Growth Form | 2 | 0.3925 | 15.6735 | < 0.001 |
| Elevation | 1 | 0.0994 | 7.9372 | < 0.001 |
| Residual | 340 | 4.2573 |  |  |

Table S3

Standardized major axes (SMA) regression parameters of the Stoichiometric scaling relationship between nitrogen (N) and phosphorus (P) concentrations, and the comparison between bryophyte in different habitats (Aquatic vs Terrestrial) as well as with different growth forms (Orthotrop vs Plagiotrop).

Values for slope, intercept and their confidence interval, coefficient of determination (*R*^2^) and *P* values of the SMA regression were shown in the table. The *P*_S_ and *P*_I_ represent the significances of slope and intercept between habitats and between growth forms, respectively.

| Groups | Slope (CI) | Intercept (CI) | *R*^2^ | *P* | *P*_S_ | *P*_I_ |
| --- | --- | --- | --- | --- | --- | --- |
| All | 0.60 (0.55-0.65) | 1.17 (1.15-1.18) | 0.15 | <0.001 | - | - |
| Aquatic | 0.41 (0.37-0.45) | 1.27 (1.25-1.29) | 0.36 | <0.001 | <0.001 | <0.001 |
| Terrestrial | 0.68 (0.61-0.77) | 1.05 (1.02-1.07) | 0.25 | <0.001 |  |  |
| All | 0.57 (0.52-0.62) | 1.17 (1.15-1.18) | 0.15 | <0.001 | - | - |
| Orthotrop | 0.54 (0.47-0.62) | 1.16 (1.13-1.19) | 0.10 | <0.001 | 0.321 | 0.573 |
| Plagiotrop | 0.59 (0.53-0.66) | 1.17 (1.15-1.19) | 0.13 | <0.001 |  |  |

Appendix 1 Materials and methods

*1.1. Data collection*

We searched Web of Science (<https://www.webofscience.com/>) and China National Knowledge Infrastructure (<https://www.cnki.net/>) for articles that were published before May 25, 2021. The keywords used in the searches were (a) “bryophyte” or “liverwort” or “moss” and (b) “stoichiometry” or (“nitrogen” and “phosphorus”) or (“carbon” and “nitrogen”) or (“carbon” and “phosphorus”).

Concentrations of C, N, P and their ratios for bryophytes were extracted from these published studies. Values were extracted from tables or figures by using WebPlotDigitizer (<https://apps.automeris.io/wpd/>). When the stoichiometric ratio was not shown, we calculated the molar ratios from the concentration of the elements. The latitude, longitude, mean annual temperature (MAT), and mean annual precipitation (MAP) for each study site were also extracted. For the articles without MAT and MAP, we extracted these variables from the WorldClim database (<https://www.worldclim.org>) by acquiring the highest resolution climate data for each site location.

Habitat conditions for bryophytes were acquired and then sorted into two categories: (i) aquatic habitats in which bryophytes species in direct contact with continuous water flow, such as springs; and (ii) terrestrial habitats, such as forests, tundra, and peatlands. Terrestrial bryophytes were further sorted into closed habitats (e.g. forests), open habitats (e.g. tundra) and peatland (e.g. bogs and fens). Bryophyte species were sorted into two growth forms according to whether their shoots stand vertically (orthotrop) or lie horizontally on the substrate (plagiotrop). In addition, life forms were used to characterize bryophytes species [1]. Bryophytes were sorted into 6 main life forms: cushion, dendroid, mat, turf, weft, and pendent.

Studies that reported results of manipulated experiments, such as experimental warming and nutrient enrichment, were also considered, yet only the values from controls that represent the bryophyte condition under ambient environment were included in our dataset. A total of 645 observations was acquired. These observations spanned latitudes from 60.7°S to 80.9°N, MAT from -20.1 to 24.7°C, and MAP from 26.5 to 5700 mm. See supplementary materials (Appendix 3) for the full list of references.

*1.2. Phylogenetic analysis*

We built a phylogeny covering 139 species of bryophytes found in the C, N, P concentration and ratio dataset. The phylogeny was based on the trnL gene, which is regarded as a prime candidate in DNA barcoding [2], obtained from GenBank [3] (see accession number in Appendix 4). Genes were aligned using MAFFT [4].

We fitted a mixed multivariate and phylogenetically informed model using a Bayesian framework and R [5] brms package [6]. In the model, we used C, N, P, C:N, C:P and N:P as response variables and latitude, elevation, MAT, MAP, habitat type, growth form as explanatory variables. The model was fitted using a log-normal distribution for the response variables, with species as a random factor and controlled for phylogenetic effects using the phylogenetic tree. Priors used followed a normal distribution (0, 2) for beta estimates of the predictors and a t-student distribution (3, 0, 2.5) for the intercept, random and phylogenetic effects, and the residuals. We estimated the probability of direction (PD) and Bayes factors (BFs) for all predictors as an estimation of the relevance of the explanatory. PD and BFs were based on the null hypothesis that mean = 0, using the bayestestR package [7].

Growth forms are genetically controlled characteristics [8]. To further test the effect of growth form, we fitted both phylogenetically informed and no phylogeny incorporated general linear model, in which C, N, P, C:N, C:P and N:P as response variables and growth form as the explanatory variable. The models were fitted with a subset of data, in which one record was randomly sampled for each species. We repeated the tests 999 times, and compared the effect of growth form between models that phylogeny incorporated or not. To test if each growth form presents phylogenetic signal, we then calculate the phylogenetic independent contrasts (PICs) for C, N, P, C:N, C:P and N:P. Large contrast values indicate that species are very divergent in their traits, which may suggest the lack of phylogenetic signal, while small contrast values suggest phylogenetic signal. The mean PIC has therefore been used to quantify the phylogenetic signal. Thus, the PICs were averaged for each growth form and all species. We calculated the rank of the mean PIC in the combination of the mean PIC and the null distribution. If the rank of the mean PIC is low (< 25, P < 0.025), it would be indicative of significant phylogenetic signal, since the mean PIC is lower than most random values. Conversely, if the rank of the mean PIC is large (> 975, P < 1-0.975 = 0.025), this indicates significant phylogenetic antisignal [9].

*1.3. Statistical analysis*

In addition, we performed regressions, redundancy analysis (RDA), and variance partitioning to examine the effects of habitat types, climate and growth forms, since species identities were not available for some of the data and cannot be incorporated in the phylogenetic analysis. Elemental concentrations and stoichiometric ratios were log10-transformed before all statistical analyses. We used linear, quadratic and cubic regressions to examine the relationships between spatial and climatic variables, namely latitude, elevation, MAT and MAP, and nutrient concentrations and stoichiometric ratios. The model with the lowest Akaike’s Information Criterion (AIC) is presented in the results. Because only a few (5%) observations were acquired from the sites with more than 1900 mm precipitation, additional linear regressions were fitted to examine the relations between MAP and elemental concentrations and ratios with a subset of data where MAP was less than 1900 mm. Bootstrap-t test was used to compare the differences in C, N, and P concentrations and stoichiometric ratios between habitat types (aquatic vs terrestrial) and between growth forms. One-way ANOVAs followed by Tukey’s HSD were carried out to test for differences in the elemental concentrations and ratios between bryophyte life forms. The standardized major axes approach (SMA) was used to calculate the bivariate log-log scaling relationships between N and P concentrations [10]. The differences in the SMA slopes were tested between habitats and growth forms.

We performed redundancy analysis (RDA) with the R package “vegan” to summarize the variation in C, N, and P concentrations and ratios that can be explained by independent variables, namely latitude, elevation, MAT, MAP, habitat, growth from and life from [11]. The explanatory variables were selected by using automatic stepwise model building according to permutation *P*-values (ordistep) and adjusted *R*^2^ (ordiR2step). Both functions selected the same set of explanatory variables. We used variation partitioning to quantify the explanatory power of climate, habitat, and growth form. All analyses were conducted with R v 4.0.5 [5], and all tests were considered significant when *P* < 0.05.

*References*

1. Mägdefrau K. *Life-forms of Bryophytes*. In: Smith AJE, (ed.). Bryophyte Ecology. Dordrecht: Springer, 1982, 45-58.

2. Stech M and Quandt D. 20,000 species and five key markers: The status of molecular bryophyte phylogenetics. *Phytotaxa* 2010; 10.11646/phytotaxa.9.1.11: 196-228.

3. Benson DA, Cavanaugh M, Clark K *et al.* GenBank. *Nucleic Acids Research* 2018; **46**: D41-D7.

4. Katoh K, Misawa K, Kuma Ki *et al.* MAFFT: a novel method for rapid multiple sequence alignment based on fast Fourier transform. *Nucleic Acids Research* 2002; **30**: 3059-66.

5. R Core Team. *R: A Language and Environment for Statistical Computing*. Vienna, Austria: R Foundation for Statistical Computing, 2021.

6. Bürkner P-C. brms: An R Package for Bayesian Multilevel Models Using Stan. *Journal of Statistical Software* 2017; **80**: 1 - 28.

7. Makowski D, Ben-Shachar MS and Lüdecke D. bayestestR: Describing Effects and their Uncertainty, Existence and Significance within the Bayesian Framework. *Journal of Open Source Software* 2019; **4**: 1541.

8. La Farge-England C. Growth Form, Branching Pattern, and Perichaetial Position in Mosses: Cladocarpy and Pleurocarpy Redefined. *The Bryologist* 1996; **99**: 170-86.

9. Swenson NG. *Functional and Phylogenetic Ecology in R*. New York Springer, 2014.

10. Warton DI, Wright IJ, Falster DS *et al.* Bivariate line-fitting methods for allometry. *Biol. Rev.* 2006; **81**: 259-91.

11. Oksanen J, Blanchet FG, Friendly M *et al.* vegan: Community Ecology Package. R package version 2.5-7; 2020.

Appendix 2 Summaries of phylogenetically informed model

1. Formula:

| Variables | Family | Formula |
| --- | --- | --- |
| C | lognormal | c ~ latitude + elevation + mat + map + habitat + growth_form + (1 \| gr(gen_spec, cov = A)) + (1\|sp) |
| N | lognormal | n ~ latitude + elevation + mat + map + habitat + growth_form + (1 \| gr(gen_spec, cov = A)) + (1\|sp) |
| P | lognormal | p ~ latitude + elevation + mat + map + habitat + growth_form + (1 \| gr(gen_spec, cov = A)) + (1\|sp) |
| C:N | lognormal | cn ~ latitude + elevation + mat + map + habitat + growth_form + (1 \| gr(gen_spec, cov = A)) + (1\|sp) |
| C:P | lognormal | cp ~ latitude + elevation + mat + map + habitat + growth_form + (1 \| gr(gen_spec, cov = A)) + (1\|sp) |
| N:P | lognormal | np ~ latitude + elevation + mat + map + habitat + growth_form + (1 \| gr(gen_spec, cov = A)) + (1\|sp) |

2. R^2^

|  |  | Estimate | Est.Error | Q2.5 | Q97.5 |
| --- | --- | --- | --- | --- | --- |
| R^2^ | C | 0.67 | 0.03 | 0.60 | 0.72 |
| R^2^ | N | 0.62 | 0.03 | 0.56 | 0.67 |
| R^2^ | P | 0.42 | 0.07 | 0.29 | 0.55 |
| R^2^ | C:N | 0.56 | 0.05 | 0.46 | 0.66 |
| R^2^ | C:P | 0.08 | 0.02 | 0.05 | 0.13 |
| R^2^ | N:P | 0.09 | 0.02 | 0.06 | 0.13 |

3. Parameters

| Variables | Parameters | Estimate | Est.Error | l-95% CI | u-95% CI | Rhat | Bulk_ESS | Tail_ESS | pd | pval | bf |
| --- | --- | --- | --- | --- | --- | --- | --- | --- | --- | --- | --- |
| C | Intercept | 6.32 | 0.15 | 6.03 | 6.61 | 1.00 | 128007.90 | 101946.51 | 1.0000 | 0.0000 | **4.27E+63** |
| C | latitude | -0.01 | 0.00 | -0.01 | 0.00 | 1.00 | 110986.61 | 100745.94 | 0.9966 | 0.0067 | 0.05 |
| C | elevation | 0.00 | 0.00 | 0.00 | 0.00 | 1.00 | 136580.00 | 107786.86 | 1.0000 | 0.0000 | **17.32** |
| C | mat | -0.01 | 0.00 | -0.02 | 0.00 | 1.00 | 162092.43 | 101744.98 | 0.9962 | 0.0075 | 0.06 |
| C | map | 0.00 | 0.00 | 0.00 | 0.00 | 1.00 | 102109.85 | 99177.46 | 1.0000 | 0.0000 | **2.64E+05** |
| C | habitat.L | 0.12 | 0.03 | 0.06 | 0.17 | 1.00 | 38779.45 | 68042.24 | 1.0000 | 0.0000 | **57.60** |
| C | growth_form.L | -0.04 | 0.02 | -0.08 | 0.00 | 1.00 | 63655.25 | 77564.50 | 0.9621 | 0.0758 | 0.06 |
| N | Intercept | 3.11 | 0.27 | 2.59 | 3.64 | 1.00 | 82993.48 | 66525.17 | 1.0000 | 0.0000 | **5.00E+11** |
| N | latitude | -0.01 | 0.00 | -0.02 | 0.00 | 1.00 | 80855.19 | 66439.69 | 0.9796 | 0.0408 | 0.02 |
| N | elevation | 0.00 | 0.00 | 0.00 | 0.00 | 1.00 | 93699.41 | 69876.27 | 0.8850 | 0.2301 | 3.30E-05 |
| N | mat | 0.01 | 0.01 | 0.00 | 0.02 | 1.00 | 91594.57 | 65938.82 | 0.9005 | 0.1991 | 0.01 |
| N | map | 0.00 | 0.00 | 0.00 | 0.00 | 1.00 | 93817.64 | 72798.26 | 0.9925 | 0.0151 | 3.40E-04 |
| N | habitat.L | -0.25 | 0.05 | -0.34 | -0.15 | 1.00 | 54114.64 | 59769.85 | 1.0000 | 0.0000 | **643.07** |
| N | growth_form.L | 0.08 | 0.06 | -0.03 | 0.19 | 1.00 | 48143.36 | 55055.53 | 0.9171 | 0.1657 | 0.08 |
| P | Intercept | 2.64 | 0.72 | 1.22 | 4.05 | 1.00 | 69478.48 | 63334.20 | 0.9998 | 0.0003 | 0.18 |
| P | latitude | -0.04 | 0.01 | -0.06 | -0.02 | 1.00 | 65044.72 | 61940.64 | 0.9996 | 0.0008 | **1.78** |
| P | elevation | 0.00 | 0.00 | 0.00 | 0.00 | 1.00 | 76262.45 | 67567.90 | 0.9994 | 0.0013 | 0.01 |
| P | mat | -0.05 | 0.02 | -0.08 | -0.02 | 1.00 | 72881.62 | 61527.27 | 0.9985 | 0.0030 | 0.74 |
| P | map | 0.00 | 0.00 | 0.00 | 0.00 | 1.00 | 83363.07 | 59886.54 | 0.6122 | 0.7756 | 6.68E-05 |
| P | habitat.L | 0.23 | 0.11 | 0.01 | 0.44 | 1.00 | 59338.83 | 60830.66 | 0.9816 | 0.0369 | 0.49 |
| P | growth_form.L | 0.13 | 0.11 | -0.10 | 0.34 | 1.00 | 32326.06 | 50196.01 | 0.8724 | 0.2552 | 0.11 |
| C:N | Intercept | 2.95 | 0.42 | 2.13 | 3.77 | 1.00 | 78730.74 | 60460.16 | 1.0000 | 0.0000 | **1325.36** |
| C:N | latitude | 0.01 | 0.01 | 0.00 | 0.02 | 1.00 | 69166.14 | 62682.06 | 0.9296 | 0.1409 | 0.01 |
| C:N | elevation | 0.00 | 0.00 | 0.00 | 0.00 | 1.00 | 96215.56 | 69107.52 | 0.9100 | 0.1800 | 6.15E-05 |
| C:N | mat | -0.02 | 0.01 | -0.04 | 0.00 | 1.00 | 91593.53 | 65085.39 | 0.9711 | 0.0577 | 0.03 |
| C:N | map | 0.00 | 0.00 | 0.00 | 0.00 | 1.00 | 89377.90 | 67516.83 | 0.9895 | 0.0210 | 6.38E-04 |
| C:N | habitat.L | 0.13 | 0.07 | -0.01 | 0.26 | 1.00 | 62321.87 | 60886.74 | 0.9646 | 0.0709 | 0.18 |
| C:N | growth_form.L | -0.09 | 0.07 | -0.22 | 0.04 | 1.00 | 63961.57 | 59355.68 | 0.9104 | 0.1793 | 0.08 |
| C:P | Intercept | 5.51 | 1.21 | 3.15 | 7.87 | 1.00 | 57856.65 | 59710.98 | 1.0000 | 0.0000 | **6.96** |
| C:P | latitude | 0.03 | 0.02 | -0.01 | 0.07 | 1.00 | 49610.65 | 58748.25 | 0.9122 | 0.1757 | 0.03 |
| C:P | elevation | 0.00 | 0.00 | 0.00 | 0.00 | 1.00 | 98895.28 | 69922.83 | 0.7687 | 0.4626 | 8.06E-05 |
| C:P | mat | -0.01 | 0.03 | -0.06 | 0.04 | 1.00 | 72343.48 | 62454.65 | 0.6505 | 0.6990 | 0.01 |
| C:P | map | 0.00 | 0.00 | 0.00 | 0.00 | 1.00 | 74462.09 | 63870.02 | 0.7012 | 0.5976 | 1.11E-04 |
| C:P | habitat.L | -0.22 | 0.18 | -0.57 | 0.12 | 1.00 | 49933.80 | 59514.89 | 0.8972 | 0.2057 | 0.19 |
| C:P | growth_form.L | -0.14 | 0.11 | -0.35 | 0.09 | 1.00 | 49554.96 | 56760.81 | 0.8910 | 0.2180 | 0.13 |
| N:P | Intercept | 1.27 | 0.63 | 0.04 | 2.50 | 1.00 | 82820.23 | 63849.21 | 0.9786 | 0.0428 | 1.68E-03 |
| N:P | latitude | 0.03 | 0.01 | 0.01 | 0.05 | 1.00 | 76351.89 | 64969.18 | 0.9987 | 0.0026 | 0.57 |
| N:P | elevation | 0.00 | 0.00 | 0.00 | 0.00 | 1.00 | 91931.82 | 68457.79 | 0.9924 | 0.0152 | 7.70E-04 |
| N:P | mat | 0.06 | 0.02 | 0.03 | 0.09 | 1.00 | 86862.49 | 64896.38 | 1.0000 | 0.0001 | **20.38** |
| N:P | map | 0.00 | 0.00 | 0.00 | 0.00 | 1.00 | 84840.46 | 60977.51 | 0.5849 | 0.8303 | 5.95E-05 |
| N:P | habitat.L | -0.36 | 0.09 | -0.53 | -0.18 | 1.00 | 69840.16 | 63574.84 | 1.0000 | 0.0001 | **88.36** |
| N:P | growth_form.L | -0.11 | 0.08 | -0.26 | 0.06 | 1.00 | 35516.83 | 44762.89 | 0.9148 | 0.1705 | 0.12 |

Appendix 3 The literature from which data were extracted

Aerts R, Callaghan TV, Dorrepaal E, Van Logtestijn RSP, Cornelissen JHC. 2009. Seasonal climate manipulations result in species-specific changes in leaf nutrient levels and isotopic composition in a sub-arctic bog. Functional Ecology 23: 680-688.

Alster CJ, Allison SD, Treseder KK. 2020. Carbon budgets for soil and plants respond to long-term warming in an Alaskan boreal forest. Biogeochemistry 150: 345-353.

Arroniz-Crespo M, Leake JR, Horton P, Phoenix GK. 2008. Bryophyte physiological responses to, and recovery from, long-term nitrogen deposition and phosphorus fertilisation in acidic grassland. New Phytologist 180: 864-874.

Ball BA, Virginia RA. 2014. The ecological role of moss in a polar desert: implications for aboveground-belowground and terrestrial-aquatic linkages. Polar Biology 37: 651-664.

Ball BA, Guevara JA. 2015. The nutrient plasticity of moss-dominated crust in the urbanized Sonoran Desert. Plant and Soil 389: 225-235.

Beyer L, White DM, Bolter M. 2001. Soil organic matter composition, transformation, and microbial colonisation of Gelic Podzols in the coastal region of East Antarctica. Australian Journal of Soil Research 39: 543-563.

Britton AJ, Gibbs S, Fisher JM, Helliwell RC. 2019. Impacts of nitrogen deposition on carbon and nitrogen cycling in alpine Racomitrium heath in the UK and prospects for recovery. Environmental Pollution 254: 10.

Bu ZJ, Zheng XX, Rydin H, Moore T, Ma JZ. 2013. Facilitation vs. competition: Does interspecific interaction affect drought responses in Sphagnum? Basic and Applied Ecology 14: 574-584.

Chapin FS, Oechel WC, Van Cleve K, Lawrence W. 1987. The role of mosses in the phosphorus cycling of an Alaskan black spruce forest. Oecologia 74: 310-315.

Chen X, Liu WY, Song L, Li S, Wu CS, Lu HZ. 2016. Adaptation of epiphytic bryophytes in the understorey attributing to the correlations and trade-offs between functional traits. Journal of Bryology 38: 110-117.

Chiwa M, Sheppardb LJ, Leith ID, Leeson SR, Tang YS, Cape JN. 2016. Sphagnum can 'filter' N deposition, but effects on the plant and porewater depend on the N form. Science of the Total Environment 559: 113-120.

Christmas M, Whitton BA. 1998. Phosphorus and aquatic bryophytes in the Swale-Ouse river system, north-east England. 1. Relationship between ambient phosphate, internal N : P ratio and surface phosphatase activity. Science of the Total Environment 210: 389-399.

Clark KL, Nadkarni NM, Gholz HL. 1998. Growth, net production, litter decomposition, and net nitrogen accumulation by epiphytic bryophytes in a tropical montane forest. Biotropica 30: 12-23.

Cusack DF, Silver W, Mcdowell WH. 2009. Biological Nitrogen Fixation in Two Tropical Forests: Ecosystem-Level Patterns and Effects of Nitrogen Fertilization. Ecosystems 12: 1299-1315.

Davey MC. 1999. The elemental and biochemical composition of bryophytes from the maritime Antarctic. Antarctic Science 11: 157-159.

Deane-Coe KK, Mauritz M, Celis G, Salmon V, Crummer KG, Natali SM, Schuur EaG. 2015. Experimental Warming Alters Productivity and Isotopic Signatures of Tundra Mosses. Ecosystems 18: 1070-1082.

Dong YP, Liu XY, Sun XC, Song W, Zheng XD, Li R, Liu CQ. 2017. Inter-species and intra-annual variations of moss nitrogen utilization: Implications for nitrogen deposition assessment. Environmental Pollution 230: 506-515.

Dong YP, Huang H, Song W, Sun XC, Wang M, Zhang W, Wang KL, Liu CQ, Liu XY. 2019. Natural C-13 and N-15 abundance of moss-substrate systems on limestones and sandstones in a karst area of subtropical China. Catena 180: 8-15.

Du EZ, Liu XY, Fang JY. 2014. Effects of nitrogen additions on biomass, stoichiometry and nutrient pools of moss Rhytidium rugosum in a boreal forest in Northeast China. Environmental Pollution 188: 166-171.

Fernandez-Martinez M, Preece C, Corbera J, Cano O, Garcia-Porta J, Sardans J, Janssens IA, Sabater F, Penuelas J. Bryophyte C:N:P stoichiometry, biogeochemical niches and elementome plasticity driven by environment and coexistence. Ecology Letters: 12.

Gordon C, Wynn JM, Woodin SJ. 2001. Impacts of increased nitrogen supply on high Arctic heath: the importance of bryophytes and phosphorus availability. New Phytologist 149: 461-471.

Gotelli NJ, Mouser PJ, Hudman SP, Morales SE, Ross DS, Ellison AM. 2008. Geographic variation in nutrient availability, stoichiometry, and metal concentrations of plants and pore-water in ombrotrophic bogs in New England, USA. Wetlands 28: 827-840.

Granath G, Strengbom J, Rydin H. 2012. Direct physiological effects of nitrogen on Sphagnum: a greenhouse experiment. Functional Ecology 26: 353-364.

Gundale MJ, Deluca TH, Nordin A. 2011. Bryophytes attenuate anthropogenic nitrogen inputs in boreal forests. Global Change Biology 17: 2743-2753.

Gundale MJ, Bach LH, Nordin A. 2013. The impact of simulated chronic nitrogen deposition on the biomass and N-2-fixation activity of two boreal feather moss-cyanobacteria associations. Biology Letters 9: 4.

Guo YR, Zhao HL, Zuo XA, Drake S, Zhao XY. 2008. Biological soil crust development and its topsoil properties in the process of dune stabilization, Inner Mongolia, China. Environmental Geology 54: 653-662.

Hajek M, Pleskova Z, Syrovatka V, Peterka T, Laburdova J, Kintrova K, Jirousek M, Hajek T. 2014. Patterns in moss element concentrations in fens across species, habitats, and regions. Perspectives in Plant Ecology Evolution and Systematics 16: 203-218.

Hoorens B, Stroetenga M, Aerts R. 2010. Litter Mixture Interactions at the Level of Plant Functional Types are Additive. Ecosystems 13: 90-98.

Hoosbeek MR, Van Breemen N, Vasander H, Buttler A, Berendse F. 2002. Potassium limits potential growth of bog vegetation under elevated atmospheric CO2 and N deposition. Global Change Biology 8: 1130-1138.

Huang JB, Liu WY, Li S, Song L, Lu HZ, Shi XM, Chen X, Hu T, Liu S, Liu T. 2019. Ecological stoichiometry of the epiphyte community in a subtropical forest canopy. Ecology and Evolution 9: 14394-14406.

Jiroušek M, Hájek M, Bragazza L. 2011. Nutrient stoichiometry in Sphagnum along a nitrogen deposition gradient in highly polluted region of Central-East Europe. Environmental Pollution 159: 585-590.

Kox MaR, Van Den Elzen E, Lamers LPM, Jetten MSM, Van Kessel M. 2020. Microbial nitrogen fixation and methane oxidation are strongly enhanced by light in Sphagnum mosses. Amb Express 10: 11.

Kracht O, Gleixner G. 2000. Isotope analysis of pyrolysis products from Sphagnum peat and dissolved organic matter from bog water. Organic Geochemistry 31: 645-654.

Kruk M, Podbielska K. 2018. Potential changes of elemental stoichiometry and vegetation production in an ombrotrophic peatland in the condition of moderate nitrogen deposition. Aquatic Botany 147: 24-33.

Li S, Liu WY, Li DW, Li ZX, Song L, Chen K, Fu Y. 2014. Slower rates of litter decomposition of dominant epiphytes in the canopy than on the forest floor in a subtropical montane forest, southwest China. Soil Biology & Biochemistry 70: 211-220.

Liu JG, Liu WG, Long XE, Chen YG, Huang TW, Huo JS, Duan LC, Wang XY. 2020a. Effects of nitrogen addition on C:N:P stoichiometry in moss crust-soil continuum in the N-limited Gurbantunggut Desert, Northwest China. European Journal of Soil Biology 98: 10.

Liu X-Y, Xiao H-Y, Liu C-Q, Li Y-Y, Xiao H-W. 2008. Stable carbon and nitrogen isotopes of the moss Haplocladium microphyllum in an urban and a background area (SW China): The role of environmental conditions and atmospheric nitrogen deposition. Atmospheric Environment 42: 5413-5423.

Liu X-Y, Koba K, Makabe A, Li X-D, Yoh M, Liu C-Q. 2013. Ammonium first: natural mosses prefer atmospheric ammonium but vary utilization of dissolved organic nitrogen depending on habitat and nitrogen deposition. New Phytologist 199: 407-419.

Liu X, Wang Z, Li XM, Bao WK. 2020b. Nitrogen and phosphorus translocation of forest floor mosses as affected by a pulse of these nutrients. Journal of Plant Ecology 13: 633-640.

Liu XY, Xiao HY, Liu CQ, Li YY, Xiao HW, Wang YL. 2010. Response of stable carbon isotope in epilithic mosses to atmospheric nitrogen deposition. Environmental Pollution 158: 2273-2281.

Ma JZ, Bu ZJ, Zheng XX, Ge JL, Wang SZ. 2015. Effects of shading on relative competitive advantage of three species of Sphagnum. Mires and Peat 16: 17.

Manninen S, Sassi MK, Loven K. 2013. Effects of nitrogen oxides on ground vegetation, Pleurozium schreberi and the soil beneath it in urban forests. Ecological Indicators 24: 485-493.

Manninen S, Kivimaki S, Leith ID, Leeson SR, Sheppard LJ. 2016. Nitrogen deposition does not enhance Sphagnum decomposition. Science of the Total Environment 571: 314-322.

Mao R, Zhang XH, Song CC, Wang XW, Finnegan PM. 2018. Plant functional group controls litter decomposition rate and its temperature sensitivity: An incubation experiment on litters from a boreal peatland in northeast China. Science of the Total Environment 626: 678-683.

Niinemets U, Kull K. 2005. Co-limitation of plant primary productivity by nitrogen and phosphorus in a species-rich wooded meadow on calcareous soils. Acta Oecologica-International Journal of Ecology 28: 345-356.

Osono T, Ueno T, Uchida M, Kanda H. 2012. Abundance and diversity of fungi in relation to chemical changes in arctic moss profiles. Polar Science 6: 121-131.

Pawlikowski P, Abramczyk K, Szczepaniuk A, Kozub L. 2013. Nitrogen:phosphorus ratio as the main ecological determinant of the differences in the species composition of brown-moss rich fens in north-eastern Poland. Preslia 85: 349-367.

Phuyal M, Artz RRE, Sheppard L, Leith ID, Johnson D. 2008. Long-term nitrogen deposition increases phosphorus limitation of bryophytes in an ombrotrophic bog. Plant Ecology 196: 111-121.

Riis T, Sandjensen K. 1997. Growth reconstruction and photosynthesis of aquatic mosses: Influence of light, temperature and carbon dioxide at depth. Journal of Ecology 85: 359-372.

Rousk K, Rousk J, Jones DL, Zackrisson O, Deluca TH. 2013. Feather moss nitrogen acquisition across natural fertility gradients in boreal forests. Soil Biology & Biochemistry 61: 86-95.

Sardans J, Penuelas J. 2008. Drought changes nutrient sources, content and stoichiometry in the bryophyte Hypnum cupressiforme Hedw. growing in a Mediterranean forest. Journal of Bryology 30: 59-65.

Scott DL, Bradley RL, Bellenger JP, Houle D, Gundale MJ, Rousk K, Deluca TH. 2018. Anthropogenic deposition of heavy metals and phosphorus may reduce biological N-2 fixation in boreal forest mosses. Science of the Total Environment 630: 203-210.

Shi WQ, Wang GA, Han WX. 2012. Altitudinal Variation in Leaf Nitrogen Concentration on the Eastern Slope of Mount Gongga on the Tibetan Plateau, China. Plos One 7: 6.

Sparrow EB, Sparrow SD, Cochran VL. 1993. PHOSPHORUS AND NITROGEN DYNAMICS DURING FIELD INCUBATIONS IN FOREST AND FALLOW SUB-ARCTIC SOILS. Biology and Fertility of Soils 16: 243-248.

Startsev NA, Lieffers VJ. 2007. Emission of nitrogen gas, nitrous oxide, and carbon dioxide on rehydration of dry feathermosses. Soil Science Society of America Journal 71: 214-218.

Sun SQ, He G, Wu YH, Zhou J, Yu D. 2013. STARCH AND NUTRIENT CONTENTS ARE KEY FOR MOSSES ADAPTING TO DIFFERENT SUCCESSION STAGES ALONG A RECEDING GLACIER. Polish Journal of Ecology 61: 233-239.

Tobias M, Niinemets U. 2010. Acclimation of photosynthetic characteristics of the moss Pleurozium schreberi to among-habitat and within-canopy light gradients. Plant Biology 12: 743-754.

Toet S, Cornelissen JHC, Aerts R, Van Logtestijn RSP, De Beus M, Stoevelaar R. 2006. Moss responses to elevated CO2 and variation in hydrology in a temperate lowland peatland. Plant Ecology 182: 27-40.

Turner BL, Baxter R, Ellwood NTW, Whitton BA. 2003. Seasonal phosphatase activities of mosses from Upper Teesdale, northern England. Journal of Bryology 25: 189-200.

Ueno T, Osono T, Kanda H. 2009. Inter- and intraspecific variations of the chemical properties of high-Arctic mosses along water-regime gradients. Polar Science 3: 134-138.

Vingiani S, Adamo P, Giordano S. 2004. Sulphur, nitrogen and carbon content of Sphagnum capillifolium and Pseudevernia furfuracea exposed in bags in the Naples urban area. Environmental Pollution 129: 145-158.

Wagner S, Zotz G, Salazar Allen N, Bader MY. 2013. Altitudinal changes in temperature responses of net photosynthesis and dark respiration in tropical bryophytes. Annals of Botany 111: 455-465.

Waite M, Sack L. 2010. How does moss photosynthesis relate to leaf and canopy structure? Trait relationships for 10 Hawaiian species of contrasting light habitats. New Phytologist 185: 156-172.

Waite M, Sack L. 2011. Does global stoichiometric theory apply to bryophytes? Tests across an elevation x soil age ecosystem matrix on Mauna Loa, Hawaii. Journal of Ecology 99: 122-134.

Wang J-N, Shi F-S, Xu B, Wang Q, Wu Y, Wu N. 2014a. Uptake and recovery of soil nitrogen by bryophytes and vascular plants in an alpine meadow. Journal of Mountain Science 11: 475-484.

Wang M, Moore TR. 2014. Carbon, Nitrogen, Phosphorus, and Potassium Stoichiometry in an Ombrotrophic Peatland Reflects Plant Functional Type. Ecosystems 17: 673-684.

Wang M, Larmola T, Murphy MT, Moore TR, Bubier JL. 2016. Stoichiometric response of shrubs and mosses to long-term nutrient (N, P and K) addition in an ombrotrophic peatland. Plant and Soil 400: 403-416.

Wang Z, Bao WK, Feng DF, Lin HH. 2014b. Functional trait scaling relationships across 13 temperate mosses growing in wintertime. Ecological Research 29: 629-639.

Wang Z, Pi CY, Li XM, Bao WK. 2019. Elevational patterns of carbon, nitrogen and phosphorus in understory bryophytes on the eastern slope of Gongga Mountain, China. Journal of Plant Ecology 12: 781-786.

Wang Z, Bader MY, Liu X, Zhu ZM, Bao WK. 2017. Comparisons of photosynthesis-related traits of 27 abundant or subordinate bryophyte species in a subalpine old-growth fir forest. Ecology and Evolution 7: 7454-7461.

Wiedermann MM, Gunnarsson U, Ericson L, Nordin A. 2009. Ecophysiological adjustment of two Sphagnum species in response to anthropogenic nitrogen deposition. New Phytologist 181: 208-217.

Williams BL, Silcock DJ. 2001. Does nitrogen addition to raised bogs influence peat phosphorus pools? Biogeochemistry 53: 307-321.

Williams BL, Buttler A, Grosvernier P, Francez AJ, Gilbert D, Ilomets M, Jauhiainen J, Matthey Y, Silcock DJ, Vasander H. 1999. The fate of NH4NO3 added to Sphagnum magellanicum carpets at five European mire sites. Biogeochemistry 45: 73-93.

Zheng MH, Chen H, Li DJ, Luo YQ, Mo JM. 2020. Substrate stoichiometry determines nitrogen fixation throughout succession in southern Chinese forests. Ecology Letters 23: 336-347.

Zheng MH, Zhang W, Luo YQ, Li DJ, Wang SH, Huang J, Lu XK, Mo JM. 2018. Stoichiometry controls asymbiotic nitrogen fixation and its response to nitrogen inputs in a nitrogen-saturated forest. Ecology 99: 2037-2046.

Zibulski R, Wesener F, Wilkes H, Plessen B, Pestryakova LA, Herzschuh U. 2017. C / N ratio, stable isotope (delta C-13, delta N-15), and n-alkane patterns of brown mosses along hydrological gradients of low-centred polygons of the Siberian Arctic. Biogeosciences 14: 1617-1630.

Zotz G, Kahler H. 2007. A moss "canopy" - Small-scale differences in microclimate and physiological traits in Tortuld ruralis. Flora 202: 661-666.

Bao WK, Lei B, Leng L. 2005. Biomass and carbon storage of ground bryophytes under six types of young coniferous forest plantations. Chinese Journal of Applied Ecology 16: 1817-1821. In Chinese with English abstract. (包维楷, 雷波, 冷俐. 2005. 六种人工针叶幼林下地表苔藓植物生物量与碳贮量. 应用生态学报 16: 1817-1821.)

Cheng GW, Luo J. 2003. The Carbon Accumulation and Dissipation Features of Sub-alpine Woodland in Mt. Gongga. Acta Geographica Sinica 58(2): 179-185. In Chinese with English abstract. (程根伟, 罗辑. 2003. 贡嘎山亚高山林地碳的积累与耗散特征. 地理学报 58: 179-185.)

Ge JL. 2016. Competition-allelopathy of three *Sphagnum* species in Changbai Mountains. Dissertations. Northeast Normal University. In Chinese with English abstract. (葛佳丽. 2016. 长白山哈泥泥炭地三种苔藓植物的竞争与化感作用. 硕士, 东北师范大学.)

Hao ZQ,Ye J, Jiang P, Lin F. 2005. Roles of bryophyte in nutrient cycling in dark coniferous forest of Changbai Mountains. Chinese Journal of Applied Ecology 16: 2263-2266. In Chinese with English abstract. (郝占庆, 叶吉, 姜萍, 蔺菲. 2005. 长白山暗针叶林苔藓植物在养分循环中的作用. 应用生态学报 16: 2263-2266.)

Hu XF. 2018. Effects of Drought Stress and Interspecific Competition on Growth and Physiological Characteristics of Two Sphagnum Species. Dissertations. Northeast Normal University. In Chinese with English abstract. (胡雪凤. 2018. 干旱胁迫和种间竞争对两种泥炭藓生长和生理特征的影响. 硕士, 东北师范大学.)

Liu BY, Liu WQ, Lei CY, Zhang YS. 2007. Physiological responses of three bryophyte species of south china to simulated nitrogen deposition. Chinese journal of plant ecology 33: 141-149. In Chinese with English abstract. (刘滨扬, 刘蔚秋, 雷纯义, 张以顺. 2007. 三种苔藓植物对模拟N沉降的生理响应. 植物生态学报 33: 141-149.)

Liu XY, Xiao HY, Liu CQ, Li YY. 2007. Contents and isotopic composition of C and N in moss(haplocladium microphyllum)tissues and soil rhizosphere. Chinese journal of plant ecology 31: 1168-1173. In Chinese with English abstract. (刘学炎, 肖化云, 刘丛强, 李友谊. 2007. 苔藓新老组织及其根际土壤的碳氮元素含量和同位素组成(δ~(13)C和δ~(15)N)对比. 植物生态学报 31: 1168-1173.)

Liu XY, Xiao HY, Liu CQ, Li YY, Xiao HW. 2009. Responses of Tissue Carbon and δ13C in Epilithic Mosses to the Variations of Anthropogenic CO2 and Atmospheric Nitrogen Deposition in City Area. Environmental Science 30: 23-28. In Chinese with English abstract. (刘学炎, 肖化云, 刘丛强, 李友谊, 肖红伟. 2009. 石生苔藓碳含量和碳同位素对城市地区人为二氧化碳和大气氮沉降变化的响应. 环境科学 30: 23-28.)

Luo L. 2012. Response of Nitrogen Contents and Nitrogen Isotopes in Mosses and Vascular Plants to the Atmosphere Nitrogen Deposition. Dissertations. Nanchang University. In Chinese with English abstract. (罗笠. 2012. 苔藓和维管束植物叶氮含量和氮同位素组成对大气氮沉降的响应. 硕士, 南昌大学.)

Tang GQ. 2018. Effect of gaps and growth substrates on epixylic bryophyte community in a Minjiang fir forest. Dissertations. Sichuan Agricultural University. In Chinese with English abstract. (汤国庆. 2018. 岷江冷杉森林林窗和生长基质对苔藓植物群落的影响. 硕士, 四川农业大学.)

Wang JY, Che KJ, Jiang ZR. 2000. A study on carbon balance of *Picea* *crassifolia* in Qilian mountains. Journal of Northwest Forestry University 15: 9-14. In Chinese with English abstract. (王金叶, 车克钧, 蒋志荣. 2000. 祁连山青海云杉林碳平衡研究. 西北林学院学报: 9-14.)

Xiao HY, Liu XY, Liu CQ. 2011. Tissue N contents and isotopic ratios in epilithic mosses indicating N deposition and transport in the atmosphere. Bulletin of Mineralogy, Petrology and Geochemistry 30: 18-25. In Chinese with English abstract. ( (肖化云, 刘学炎, 刘丛强. 2011. 石生苔藓组织氮含量和氮同位素指示贵阳地区大气氮沉降与迁移的研究. 矿物岩石地球化学通报. 30: 18-25.)

Xie ZY, Xiao HY, Zhu RG, Wu DS. 2011. Nitrogen concentrations and stable isotope in epilithic mosses to investigate atmospheric N deposition and N sources in Jiangxi Province. Chinese Journal of Environmental Science 32: 943-948. In Chinese with English abstract. (谢志英, 肖化云, 朱仁果, 吴代赦. 2011. 利用石生苔藓氮含量与氮同位素探讨江西省大气氮沉降量和来源. 环境科学32: 943-948.)

Zhang P, Ding ZJ, Hou YY, Bai HY, Ren XQ, Zhao P. 2016. Distribution of carbon isotope composition of epilithic mosses and their environmental significance in the Mount Taibai, China. Arid Zone Research 33: 1046-1056. In Chinese with English abstract. (张普, 丁宗巨, 侯瑶瑶, 白红英, 任晓倩, 赵佩. 2016. 太白山石生苔藓碳同位素分布特征及其环境指示意义. 干旱区研究 33: 1046-1056.)

Zhou XL. 2019. Monitoring the atmospheric heavy metals and nitrogen deposition by using the moss *Haplocladium* *microphyllum* (Hedw.)Broth. Doctoral Dissertations. Nanjing Forestry University. In Chinese with English abstract. (周晓丽. 2019. 利用细叶小羽藓监测大气重金属及氮沉降的研究. 博士, 南京林业大学.)

Appendix 4 Species covered in phylogeny analysis and GenBank accession numbers for the trnL gene.

| Species | Accession numbers |
| --- | --- |
| *Actinothuidium hookeri* | KF770502 |
| *Amblystegium serpens* | AY009827 |
| *Anomodon viticulosus* | AM990343 |
| *Apopellia endiviifolia* | GQ428075 |
| *Aulacomnium palustre* | FJ628078 |
| *Aulacomnium turgidum* | FJ572461 |
| *Bartramia halleriana* | AY532395 |
| *Brachythecium austro-salebrosum* | AF161119 |
| *Brachythecium buchananii* | KF434265 |
| *Brachythecium rivulare* | AM990348 |
| *Brachythecium velutinum* | GQ428067 |
| *Bryum argenteum* | LC270624 |
| *Bryopteris filicina* | AY463549 |
| *Bryum pseudotriquetrum* | AY150357 |
| *Calliergon cordifolium* | AY009836 |
| *Calliergonella cuspidata* | MH613583 |
| *Calliergon giganteum* | AY009834 |
| *Calliergon sarmentosum* | JF709993 |
| *Campylium stellatum* | MK313974 |
| *Chorisodontium aciphyllum* | MG077053 |
| *Climacium dendroides* | MW026624 |
| *Conocephalum conicum* | AY688806 |
| *Cratoneuron filicinum* | AY009826 |
| *Ctenidium molluscum* | FM211895 |
| *Dialytrichia mucronata* | GU953735 |
| *Dicranum scoparium* | KF424001 |
| *Didymodon tophaceus* | JN968449 |
| *Drepanocladus uncinatus* | HQ452095 |
| *Entodon concinnus* | MH613600 |
| *Eucladium verticillatum* | AY950402 |
| *Eurhynchium laxirete* | GU563727 |
| *Eurohypnum leptothallum* | MH613602 |
| *Fissidens grandifrons* | OL516380 |
| *Fissidens taxifolius* | OL516376 |
| *Fontinalis antipyretica* | AF191531 |
| *Frullania mirabilis* | FJ380375 |
| *Gymnostomum calcareum* | KX176744 |
| *Hamatocaulis lapponicus* | AY626008 |
| *Hamatocaulis vernicosus* | MG952384 |
| *Haplocladium microphyllum* | KX776106 |
| *Herbertus divergens* | DQ293974 |
| *Heterophyllium affine* | MK313991 |
| *Homaliodendron flabellatum* | FM210290 |
| *Homaliodendron scalpellifolium* | FM210292 |
| *Hylocomium splendens* | AF397840 |
| *Hypnum callichroum* | MK313998 |
| *Hypnum cupressiforme* | MH613631 |
| *Hypnum jutlandicum* | MH613658 |
| *Hypnum pallescens* | AY009844 |
| *Hypnum plumaeforme* | MK314021 |
| *Hypnum sakuraii* | MK314025 |
| *Lepidopilum polytrichoides* | AY306772 |
| *Lepidozia reptans* | AY608133 |
| *Leptodictyum riparium* | MH613698 |
| *Leucobryum antillarum* | KY619014 |
| *Lindbergia sinensis* | KF770506 |
| *Lophocolea bidentata* | AY149862 |
| *Lunularia cruciata* | AY688810 |
| *Meesia triquetra* | AY501419 |
| *Mnium spinosum* | JF277354 |
| *Octoblepharum pulvinatum* | MK371355 |
| *Oncophorus wahlenbergii* | MZ014509 |
| *Orthomnion dilatatum* | LC270549 |
| *Orthostichopsis tetragona* | AY306795 |
| *Oxyrrhynchium speciosum* | DQ208201 |
| *Palustriella commutata* | AF260912 |
| *Palustriella commutata* var. *falcata* | AY626006 |
| *Paraleucobryum enerve* | AF231184 |
| *Philonotis caespitosa* | KC111119 |
| *Philonotis fontana* | EF107538 |
| *Pilotrichum bipinnatum* | AY306810 |
| *Plagiomnium acutum* | LC270550 |
| *Plagiothecium handelii* | HQ665384 |
| *Plagiomnium japonicum* | LC270552 |
| *Plagiothecium nemorale* | KJ681376 |
| *Platyhypnidium riparioides* | AF397847 |
| *Plagiomnium undulatum* | GQ428078 |
| *Pleuroziopsis ruthenica* | JF828576 |
| *Pleurozium schreberi* | MH613702 |
| *Pogonatum cirratum* subsp.*fuscatum* | GU569707 |
| *Pogonatum microstomum* | GU569712 |
| *Pohlia melanodon* | JF277342 |
| *Polytrichastrum alpinum* | MF180632 |
| *Polytrichum commune* | AF478317 |
| *Polytrichum juniperinum* | FJ572490 |
| *Polytrichastrum papillatum* | GU569744 |
| *Polytrichum strictum* | MF180608 |
| *Pseudoscleropodium purum* | AF397797 |
| *Ptilium crista-castrensis* | MH613706 |
| *Racomitrium lanuginosum* | MN239148 |
| *Reboulia hemisphaerica* | GQ910702 |
| *Rhizomnium nudum* | LC270616 |
| *Rhizomnium punctatum* | JF277357 |
| *Rhodobryum ontariense* | MH175923 |
| *Rhynchostegium riparioides* | GU552294 |
| *Rhytidium rugosum* | MK314044 |
| *Rhytidiadelphus squarrosus* | AF161126 |
| *Rhynchostegiella teneriffae* | FJ262437 |
| *Rhytidiadelphus triquetrus* | HQ443882 |
| *Riccardia chamedryfolia* | KX512021 |
| *Sanionia uncinata* | HQ452095 |
| *Scorpiurium circinatum* | AF397834 |
| *Scorpidium cossonii* | MT590362 |
| *Scorpidium scorpioides* | AY626014 |
| *Sphagnum angustifolium* | AY298005 |
| *Sphagnum balticum* | AF192594 |
| *Sphagnum capillifolium* | AY298039 |
| *Sphagnum contortum* | AF192636 |
| *Sphagnum cuspidatum* | JQ712988 |
| *Sphagnum fallax* | KR067392 |
| *Sphagnum fimbriatum* | JN000135 |
| *Sphagnum flexuosum* | AY298097 |
| *Sphagnum fuscum* | JN000121 |
| *Sphagnum girgensohnii* | MG586082 |
| *Sphagnum junghuhnianum* | AY298145 |
| *Sphagnum magellanicum* | KU183770 |
| *Sphagnum ovatum* | KC682045 |
| *Sphagnum palustre* | MF362362 |
| *Sphagnum papillosum* | AY298207 |
| *Sphagnum perichaetiale* | KF864550 |
| *Sphagnum recurvum* | JQ712987 |
| *Sphagnum russowii* | AY298262 |
| *Sphagnum squarrosum* | JN000139 |
| *Sphagnum subnitens* | AY298303 |
| *Sphagnum subsecundum* | GQ370693 |
| *Sphagnum teres* | JN000140 |
| *Sphagnum warnstorfii* | AY298351 |
| *Stictolejeunea squamata* | DQ987404 |
| *Syrrhopodon incompletus* | AF231181 |
| *Thamnobryum alopecurum* | FM201513 |
| *Thuidium cymbifolium* | KF770482 |
| *Thuidium delicatulum* | AF161132 |
| *Thuidium kanedae* | KF770484 |
| *Tomentypnum nitens* | AY009854 |
| *Tortula ruralis* | AF023722 |
| *Trichostomum crispulum* | AY950436 |
| *Trichocolea tomentella* | AY456277 |
| *Warnstorfia exannulata* | DQ404994 |
| *Zelometeorium patulum* | AY306851 |
